# Reassessing the Evolutionary History of the 17q21 Inversion Polymorphism

**DOI:** 10.1093/gbe/evv214

**Published:** 2015-11-11

**Authors:** Joao M. Alves, Ana C. Lima, Isa A. Pais, Nadir Amir, Ricardo Celestino, Giovanna Piras, Maria Monne, David Comas, Peter Heutink, Lounès Chikhi, António Amorim, Alexandra M. Lopes

**Affiliations:** ^1^Doctoral Program in Areas of Basic and Applied Biology (GABBA), University of Porto, Portugal; ^2^Instituto de Investigação e Inovação em Saúde, Universidade do Porto, Portugal; ^3^Instituto de Patologia e Imunologia Molecular da Universidade do Porto—IPATIMUP, Portugal; ^4^Instituto Gulbenkian de Ciência (IGC), Oeiras, Portugal; ^5^Department of Genetics, Washington University School of Medicine, St. Louis; ^6^Laboratoire de Biochimie Appliquée, Faculté des Sciences de la Nature et de la Vie, Université Abedrrahmane Mira de Bejaia, Algerie; ^7^School of Allied Health Technologies, Polytechnic of Porto, Porto, Portugal; ^8^Department of Hematology, Centro di Diagnostica Biomoleculare et Citogenetica Emato-Oncologica, San Francesco Hospital-ASL, Nuoro, Italy; ^9^Departament de Ciències Experimentals i de la Salut, Institut de Biologia Evolutiva (CSIC-UPF), Universitat Pompeu Fabra, Barcelona, Spain; ^10^German Center for Neurodegenerative Diseases (DZNE), Tübingen, Germany; ^11^CNRS (Centre National de la Recherche Scientifique), Université Paul Sabatier, École Nationale de Formation Agronomique, Unité Mixte de Recherche 5174 EDB (Laboratoire Évolution & Diversit Biologique), Toulouse, France; ^12^Faculdade de Ciências da Universidade do Porto, Portugal; ^13^ Present address: Department of Biochemistry, Genetics and Immunology and Institute of Biomedical Research of Vigo (IBIV), University of Vigo, Vigo, Spain

**Keywords:** chromosomal inversions, human demography, human evolution

## Abstract

A polymorphic inversion that lies on chromosome 17q21 comprises two major haplotype families (H1 and H2) that not only differ in orientation but also in copy-number. Although the processes driving the spread of the inversion-associated lineage (H2) in humans remain unclear, a selective advantage has been proposed for one of its subtypes. Here, we genotyped a large panel of individuals from previously overlooked populations using a custom array with a unique panel of H2-specific single nucleotide polymorphisms and found a patchy distribution of H2 haplotypes in Africa, with North Africans displaying a higher frequency of inverted subtypes, when compared with Sub-Saharan groups. Interestingly, North African H2s were found to be closer to “non-African” chromosomes further supporting that these populations may have diverged more recently from groups outside Africa. Our results uncovered higher diversity within the H2 family than previously described, weakening the hypothesis of a strong selective sweep on all inverted chromosomes and suggesting a rather complex evolutionary history at this locus.

## Introduction

In contrast to early predictions from classical cytogenetic studies, a particular subtype of balanced rearrangement—chromosomal inversion—has been recently recognized as a common source of genomic variation (Kirkpatrick 2010). A striking example is the widespread polymorphic inversion that lies on human chromosome 17q21. This rearrangement spans nearly 1 Mb, involves several genes implicated in complex neurodegenerative disorders (Stefansson et al. 2005) and, due to the lack of recombination, two clearly distinct major haplotype families—H1 and H2—have been derived from its orientation (Standard and Inverted, respectively) (Stefansson et al. 2005; Zody et al. 2008; Antonacci et al. 2009; Donnelly et al. 2010).

With the advances in deep resequencing data analysis, new studies focused on the structural variability of the human genome (Sudmant et al. 2010; Campbell et al. 2011; Lopes et al. 2013) have revealed that the 17q21 segment is also considerably polymorphic at the copy-number (hereon, CN) level, adding a new layer of variability to the already complex architecture of the region. These CN changes mainly consist of two distinct duplications overlapping the *KANSL1* gene, and were initially thought to be restricted to European populations (Sudmant et al 2010). More recently, Steinberg et al. (2012) updated the frequency and distribution of these CN-polymorphisms (CNPs) surveying a much larger population panel and, using a complementary strategy between cytogenetics, comparative genomics and population genetics, demonstrated that each of these CNPs was distinctively associated with the previously reported structural haplotypes. In fact, although a long 205-kb CN duplication was polymorphic in the H1 lineage, a distinct, and shorter, 155-kb duplication was detected only in some H2 chromosomes.

From an evolutionary perspective, these CN variants are therefore believed to have arisen independently in the two haplotype families. Although it has been argued that the origin of the duplication events predates early human expansions (i.e., Out-of-Africa), the “derived” haplotypes (i.e., H1D and H2D), enriched in duplicated copies, are found at much higher frequencies in present-day non-African populations (Sudmant et al. 2010; Boettger et al. 2012; Steinberg et al. 2012). As a consequence, new hypotheses regarding the evolutionary history of the region emerged, placing these CNPs as potential candidates for a selective advantage. This is particularly evident in European populations, considering that the inversion-associated haplotype carrying a duplicated copy of the short CNP—H2D—is present at high frequencies (up to 25%) and displays very low levels of genetic diversity (Boettger et al. 2012; Steinberg et al. 2012).

Nevertheless, the processes driving the spread of the duplication-specific haplotypes in the human lineage remain unclear, as the observed patterns could also be the result of the complex demographic history of populations, without the need of invoking selection (Zody et al. 2008; Donnelly et al. 2010; Steinberg et al. 2012).

In this study, we propose to further explore the evolutionary history of the 17q21 region by focusing on the inversion-associated haplotype family (i.e., H2). By extending sampling efforts to previously overlooked populations, we characterized the distribution of both H2 subtypes—H2′ and H2D—in a wide panel of individuals, targeting specific geographical groups, to better understand the evolutionary processes that have shaped the 17q21 rearrangement. Moreover, the diversity patterns within the H2 lineage were re-analyzed using a unique panel of H2-specific single nucleotide polymorphisms (SNPs), derived from publicly available resequencing data and explicitly selected for this purpose. Finally, departures of nucleotide variability patterns from neutral expectations were tested using simulated data together with full-sequence information for both major haplotype families (i.e., H1 and H2).

Overall, this work provides a finer picture of the distribution and the genetic diversity of the 17q21*-inv* and attempts to clarify important aspects of the evolution of this genomic region.

## Materials and Methods

### Available Data Sets and Samples

#### SNP Genotype Data Sets

Genotype data from several North African and European samples (*n* = 137) were retrieved from a recently published database (Henn et al. 2012; Botigué et al. 2013). Sardinian SNP data, genotyped using Affymetrix 500 K SNP array, were available at PH laboratory (*n* = 100). Publicly available genotypes from a diverse population panel were obtained from the Stanford Human Genome Diversity Project (*n* = 373). Algerian (*n* = 52) and Portuguese (*n* = 49) population SNP data were generated at LC laboratory at IGC and at AA laboratory at IPATIMUP, respectively.

Also, genotype data of individuals (*n* = 1,408) from distinct populations—CEU, FIN, GBR, TSI, IBS, GWD, YRI, MSL, ESN, LWK, BEB, GIH, ITU, PJL, and STU—were retrieved from the 1000 Genomes Project ftp (Phase III release).

#### Sequence Data

High coverage resequencing data of two H2 homozygous from Eastern Africa (NA21599) (Steinberg et al. 2012) and Central South Asia (NA20890) (Boettger et al. 2012) were available through the Sequence Read Archive. Low coverage resequencing data were obtained in BAM format from the 1000 Genomes Project (Phase III release).

#### Cell Lines

Lymphoblast cell lines from five Hapmap individuals were purchased from Coriell Cell Repository (http://www.ccr.coriell.org/, last accessed November 26, 2015).

A list of all individuals used in this study, along with the corresponding inversion and duplication status, is shown in supplementary table S1, Supplementary Material online.

### Inference of Structural Profiles

**Fig. 1.**
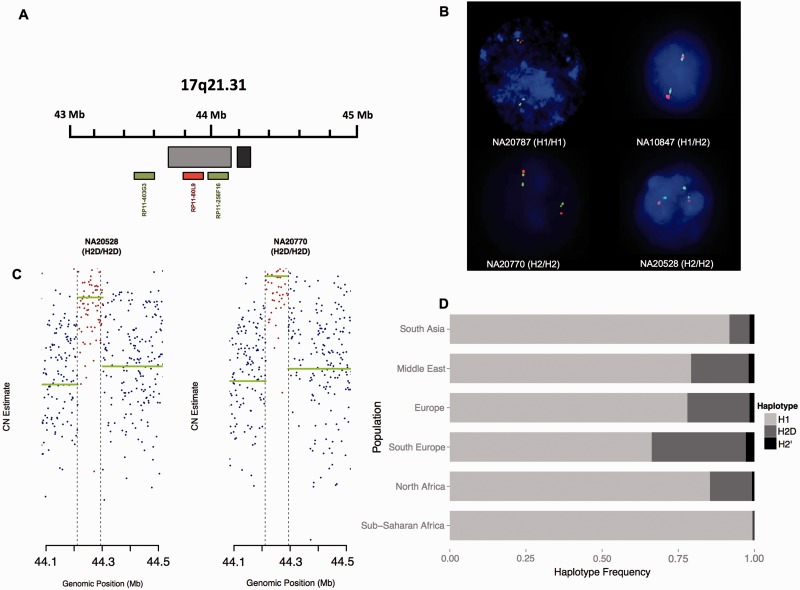
—Inference of structural profiles and cumulative frequencies of the 17q21 haplotypes in human groups. (*A*) Schematic representation of the 17q21.31 region in standard orientation. Light gray rectangle encompassing the physical location of the 17q21*-inv* (chromosome 17: 43,705,166–44,164,259, GRCh37). Dark gray rectangle encompassing the expected physical location of the H2-specific duplication—CNP155 (chromosome 17: 44,210,855–44,294,624, GRCh37). BAC probes used for dual-FISH experiments represented as green and red boxes. (*B*) Cytogenetic validation of 17q21-*inv* on four Hapmap cell lines. Green–Red–Green fluorescent-signal pattern indicates standard orientation; Green–Green–Red pattern indicates inverted orientation. SNP-derived haplotypes are shown in brackets. (*C*) Classification of copy-number profiles for the H2-specific CNP155 duplication using read-depth information of H2D/H2D (genotype-based) samples using a sliding window approach. Runs of horizontal green lines belong to the same CNV inferred. Dashed delimiters (vertical lines) specify the expected location of the CNP155 duplication. (*D*) Barplot displaying the frequency of the 17q21 structural haplotypes in human groups. Distinct colors represent each subhaplotype.

Rao et al. (2010) have found that some chromosomes identified as H2 on the basis of inversion-specific SNPs could actually carry the noninverted configuration, hence suggesting the possibility of recurrence of the inversion in the 17q21 region. We thus performed fluorescent in situ hybridization (FISH) in a small set of H2 carriers, using the same method described in Rao et al. (2010) (supplementary material and fig. S1, Supplementary Material online). Given that no incongruence was found between the FISH-determined orientation and the haplotype sequence (fig. 1), a set of previously described inversion-markers (Antonacci et al. 2009; Donnelly et al. 2010) were used to infer the inversion status for all individual samples (supplementary table S2, Supplementary Material online). Similarly, the duplication status of the H2-chromosomes was assessed mainly using two duplication-markers reported in Steinberg et al. (2012) (supplementary table S2, Supplementary Material online). Nevertheless, whenever resequenced data were available (i.e., 1000 Genomes Project data), the presence/absence of the duplication was further confirmed using the HMMCopy software (Ha et al. 2012) (fig. 1 and supplementary material and fig. S2, Supplementary Material online).

Once the orientation and duplication statuses were confirmed, each individual was grouped according to geographical origin, and six major continental groups were thus defined, Sub-Saharan Africa, North Africa, Middle East, South Asia, Central Europe, and South Europe (supplementary table S1, Supplementary Material online). Haplotype frequencies were then estimated for each geographically defined group. Note that we do not wish to give too strong a meaning to the geographical groupings above and, instead, see them as practical units devised to achieve sufficient sample sizes for the estimation of population summary statistics.

### H2-Polymorphic SNP Panel

A custom SNP genotyping assay was designed using genomic information derived from previously published data sets using two independent variant discovery strategies. First, sequence reads at high coverage from 1) an H2 homozygous from Eastern Africa (NA21599) (Steinberg et al. 2012) and 2) an H2 homozygous from Central South Asia (NA20890) (Boettger et al. 2012) were aligned to the human reference GRCh37 using BWA (Bwa-0.6.2) (Li and Durbin 2010). Following a standardized best-practices pipeline (Auwera et al. 2013), mapped reads were processed by 1) removing poorly mapped reads, 2) performing local realignment around indels, and 3) marking polymerase chain reaction duplicates. SNP calling was subsequently performed using the genome analysis toolkit Unified Genotyper (GATK-UG) algorithm (DePristo et al. 2011). The resulting VCF was then recalibrated to filter variants below required sensitivity levels.

In parallel, variant calling was also performed on multiple European H2 homozygous samples, sequenced at low-coverage (supplementary table S3, Supplementary Material online), using the GATK-UG (DePristo et al. 2011). Following software recommendations, hard filtering options using read- and variant-based parameters were then applied to remove potential false-positives.

Afterwards, the two variant sets were merged and a total of 203 previously unidentified SNPs were found to be polymorphic in at least one individual from the list. Due to the highly repetitive architecture of the 17q21 region, the SNP set was further pruned to avoid variants located within known segmental duplications (*n* = 67). The remaining SNPs (*n* = 136) were then genotyped in all H2 carriers, for which DNA was available, using SEQUENOM platform (supplementary table S1, Supplementary Material online). Moreover, 17 inversion- and duplication-tagging SNPs were added to the assay to check for genotype consistency. The quality of the obtained genotypes was then verified using Plink software (Purcell et al. 2007). Given that approximately half of the genotyped SNPs (*n* = 79) were found to be monomorphic, these variants were removed for the remaining of the study as they more likely represent false positives or singletons (supplementary table S4, Supplementary Material online). Thus, a final list of 74 SNPs were kept for subsequent analysis (genotype rate: 0.998).

### Haplotype Phasing, Genetic Differentiation, and Phylogenetic Analysis

Recent years have witnessed the development of a variety of statistical algorithms for haplotype reconstruction from unrelated genotype data (Stephens et al. 2001; Browning and Browning 2011; Delaneau et al. 2013) that generally rely on a pre-existent reference list of preassembled haplotypes to accurately generate phase information. We therefore phased the genotypes from the previous step using SHAPEIT (v2) software (Delaneau et al. 2013) and the 1000 Genomes Project data as the reference panel of haplotypes. Following the software recommendations, all genotypes were simultaneously phased for a total of 200 iterations with a burn-in and pruning stage of 50 iterations each. The obtained haplotypes were then stored in VCF format and combined with the ones from the 1000 Genomes Project (Phase III release). Afterwards, a principal component analysis was performed on all haplotypes to check for possible phasing errors (supplementary fig. S3, Supplementary Material online). Phased haplotypes were finally sorted according to the three possible statuses (i.e., H1, H2′, and H2D) and grouped by continental origin. Nucleotide diversity estimates were measured for each haplotype using VCFdivstat (v1.0). Genetic differentiation estimates were assessed between all continental pairs using adegenet R package (Jombart 2008). Finally, a Bayesian phylogenetic tree was generated using MrBayes Software (v3.2.3) (Ronquist and Huelsenbeck 2003) for most haplotypes available (but see below) for a total of 5,000,000 iterations. Samples were taken every 500 iterations.

### Neutrality Tests Using Simulation-Based Approaches

Given that our SNP panel was primarily designed to specifically capture H2 intrahaplotypic variation, and considering the heavy effects of SNP ascertainment bias in standard genetic methods of analysis, full sequence information from the 1000 Genomes Project was next used to estimate summary statistics of population genetic variation for each major haplotype family—H1 and H2. Using the ANGSD software (Korneliussen et al. 2014), nucleotide diversity estimates and Tajima’s *D* scores were independently inferred for (*) the full set of H2 homozygous samples available (*N* = 32), and (**) a subset of randomly selected H1 homozygous samples (*N* = 150) (supplementary table S1, Supplementary Material online). To assess the statistical significance of Tajima’s *D*, genetic data were then simulated under neutrality using invertFREGENE software (O’Reilly et al. 2010) for a total of 1,000 runs. As an overview, the simulation framework begins with the introduction of a 500-kb inversion in a panmictic population of constant size (*N*_e_ = 10,000). The inversion will then be transmitted to subsequent generations until a target frequency is reached. Here, the target frequency was set to 0.337—highest known frequency of the 17q21*-inv*. Afterwards, Tajima’s *D* scores were estimated from the simulated data to generate null distributions for both inverted and noninverted chromosomal types.

## Results

### Reassessing the Distribution of the 17q21 Haplotypes

After confirming that the structural profiles of the 17q21 region (i.e., inversion- and duplication-status) could be reliable inferred from previously described single nucleotide markers (Donnelly et al. 2010; Steinberg et al. 2012) (fig. 1*B* and *C*, supplementary material, Supplementary Material online), the orientation and duplication statuses at 17q21 were assessed using genotype data from 2,120 individuals belonging to several present-day populations (table 1). As expected, our results indicate that both inversion-associated subhaplotypes (i.e., H2′ and H2D) display a rather discontinuous distribution across human groups (fig. 1*D*), with cumulative frequencies varying between 0.7% in Sub-Saharan African populations and 33.7% in Southern Europeans. Contrasting with previous estimates (Steinberg et al. 2012), a cumulative frequency of 8.2% was found in South Asian populations. Nevertheless, it is worth noting that in Steinberg et al. (2012) the Asian group comprised samples from South, Central, and East Asian groups and the uneven distribution of the H2 haplotype in these populations may help explain the discrepancies found in frequency estimates. Interestingly, both subtypes are also segregating at appreciable frequencies in North Africans (14.6%). Finally, it is worth noting that, with the exception of Sub-Saharan Africans where both subtypes segregate at similar frequencies, most geographical groups show an enrichment of the duplicated H2D configuration, corroborating previous findings (Steinberg et al. 2012).

**Table 1:** Number of Individuals Used in This Study by Geographical Origin

Population	Number of Individuals
Sub-Saharan Africa	523
North Africa	181
Middle East	156
South Asia	549
Europe	350
South Europe	361
Total	2,120

### Nucleotide Diversity and Population Differentiation within the H2 Lineage

Inversion carriers were subsequently genotyped for a unique panel of newly identified H2-specific SNPs (see Materials and Methods) to further refine the diversity patterns within the 17q21 inversion-associated haplotypes. Our results uncovered appreciable levels of haplotype diversity for both inverted subtypes (table 2). These results are therefore in contrast with previous findings (Steinberg et al. 2012), where very low levels of diversity within the H2 lineage, in particular H2D, were inferred. Moreover, we found that H2D and H2′ diversity were highest in African populations, but given that these estimates were derived from a small number of chromosomes, future work in larger sample sizes may be needed to validate this result.

**Table 2:** Nucleotide Diversity Estimates for H2 Subtypes

	Nucleotide Diversity Estimates	
Population	H2D	H2′
Sub-Saharan Africa	1.32E-05	3.17E-06
North Africa	5.59E-06	7.00E-06
Middle East	4.50E-06	5.40E-06
South Asia	3.24E-06	3.49E-06
Europe	5.28E-06	5.42E-06
South Europe	6.18E-06	5.65E-06
All populations	5.64E-06	5.62E-06

**Fig. 2.**
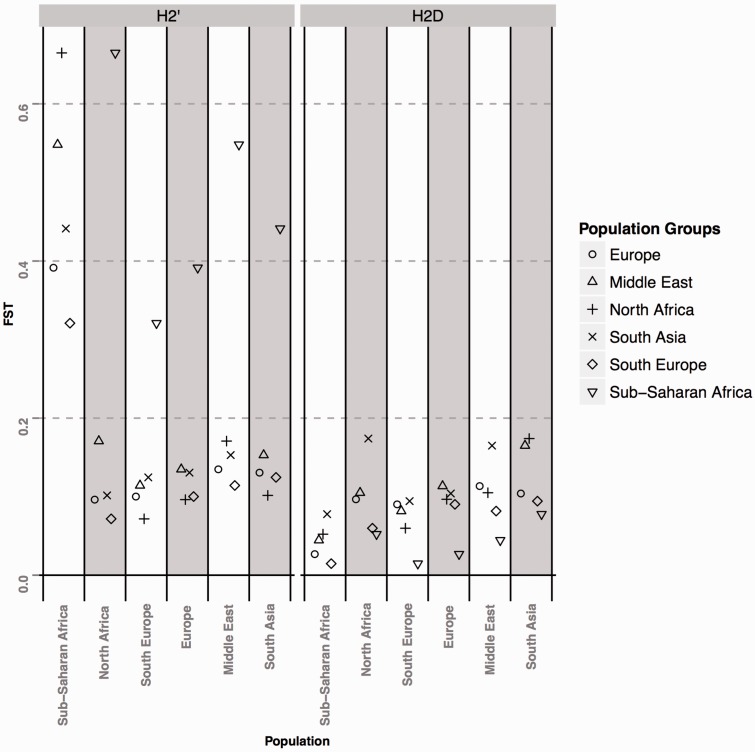
—Population stratification of H2 subtypes. Distribution of *F*_st_ values based on pairwise comparisons between all geographical regions within H2′ (left) and H2D (right) subhaplotypes. Distinct symbols represent each geographical group.

Afterwards, genetic differentiation was assessed through pairwise *F*_st_ estimates between geographical groups within each structural subtype. The results are illustrated in figure 2. Although we did not observe significant levels of population stratification within the H2D subtype (*F*_st_ < 0.20), Sub-Saharan African H2′ chromosomes are substantially different from the ones in the remaining geographical groups. Interestingly, the highest levels of genetic differentiation were observed between Sub-Saharan and North African/Middle Eastern populations, where *F*_st_ scores exceeded 0.5.

### Phylogenetic Relationship between the 17q21 Haplotypes

It is important to note that the analyses performed so far mainly rely on allele frequency information to determine whether the two inversion-associated subhaplotypes display distinct patterns of diversity and differentiation between present-day meta-population groups. Although these measures would not be affected by minor subhaplotypic phasing errors (e.g., incorrectly derived haplotypes from an H2D homozygous sample), the same would not apply when considering phylogenetic approaches, where possible errors in the phasing step would likely result in inaccurate inference of evolutionary relationships between chromosomes.

**Fig. 3.**
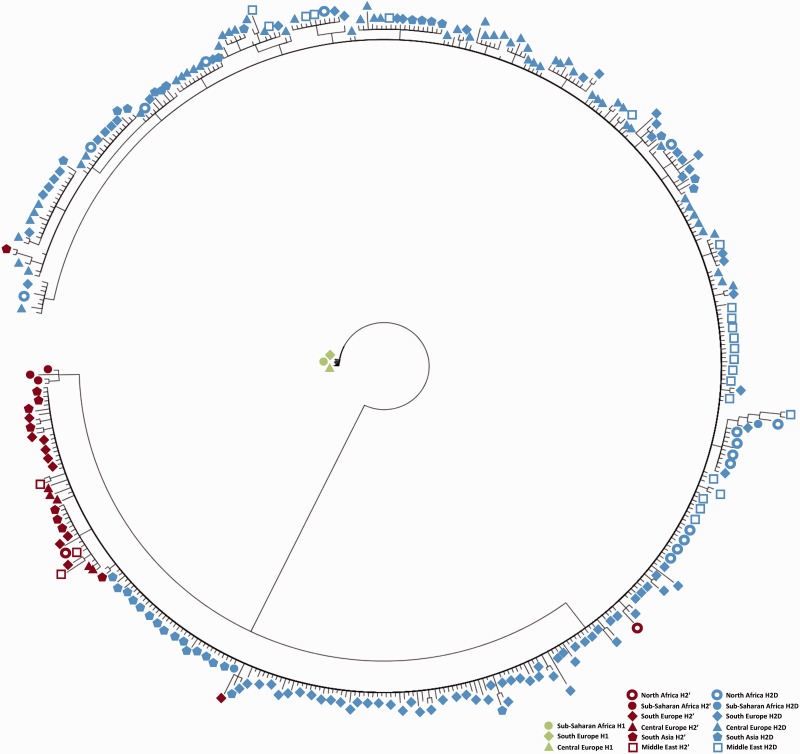
—Phylogenetic relationships between 17q21 haplotypes. Bayesian phylogenetic reconstruction through Markov Chain Monte Carlo method on all statistically derived haplotype sequences. Distinct colors represent the haplotype configuration (i.e., H1, H2′, and H2D), whereas different symbols represent each population.

To circumvent this limitation, we performed a phylogenetic analysis including only heterokaryotype individuals (i.e., individuals heterozygous for the orientation). This approach allows excluding from the analyses incorrectly phased chromosomes, which could be confounded with genetic recombinants. The phylogenetic tree obtained with the 17q21 haplotypes derived from heterokaryotypes is thus shown in figure 3.

As expected, all chromosomes belonging to the H1 lineage cluster together in a single branch, regardless of the continental origin, clearly separated from the H2 haplotypes. Interestingly, the first split corresponds to Sub-Saharan African H2′ copies whereas the second split incorporates the remaining H2′ chromosomes and all H2D copies. This suggests that the H2D evolved from an H2′ copy (or set of copies). Moreover, although there is not enough phylogenetic information to be more precise at this stage, the lack of population stratification between (or within) subtypes suggests that recombination events between inverted copies have likely occurred throughout the evolution of the 17q21 region.

### Testing Departures from Neutral Expectations

**Fig. 4.**
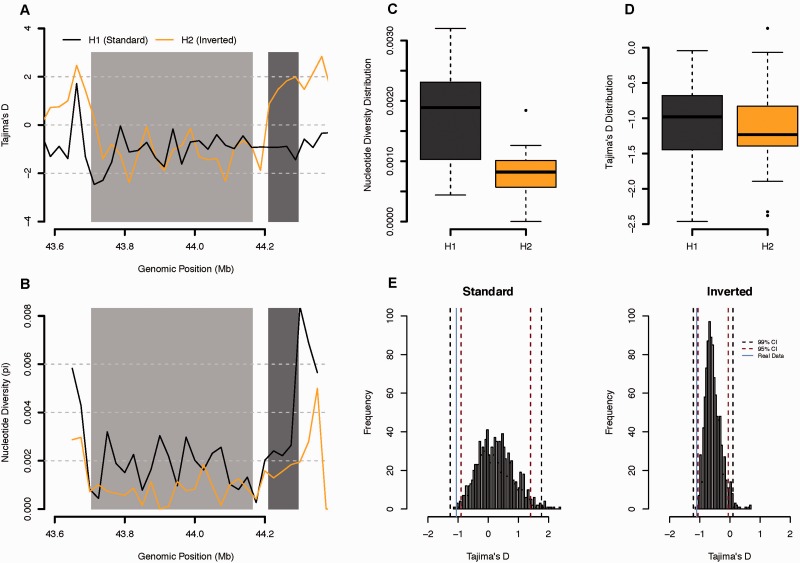
—Sliding window analysis of the interhaplotypic variation of the 17q21 major haplotype families. Distribution of nucleotide diversity (π) and Tajima’s *D* estimates obtained for the genomic region encompassing the inverted rearrangement. (*A*) Values of Tajima’s *D* estimated over genomic windows of 25 kb without overlap. Each color represents one of the major haplotype families. Light gray rectangle encompasses the genomic position covered by the 17q21*-inv.* Dark gray rectangle encompasses the expected position of the H2-specific duplication—CNP155. (*B*) Estimates of nucleotide diversity over genomic windows of 25 kb without overlap. Each color represents one of the major haplotype families. (*C*) Boxplots displaying the nucleotide diversity distribution between H1 (dark gray) and H2 (orange) haplotypes. (*D*) Boxplot displaying the Tajima’s *D* distribution between H1 (dark gray) and H2 (orange) haplotypes (*E*) Tajima’s *D* distribution from the simulated data obtained over 1,000 runs. Left and right histogram represents the Tajima’s *D* scores obtained for the standard and inverted orientation, respectively. Solid blue line depicts the mean score of Tajima’s *D* for each orientation using real data. Dashed lines present the confidence intervals calculated for 99% (black line) and 95% (red line).

As stated above, another important issue regarding the 17q21 locus is whether selection has played a major role in its evolutionary history. Although a variety of statistical tests have been devised to detect selection from SNP genotype data (Vitti et al. 2013), one important caveat in most approaches is the assumption of an unbiased ascertainment of SNPs across the genomic region under study. In consequence, a set of population summary statistics, based on levels of polymorphism, was estimated for both orientations (i.e., H1 and H2) from individuals for which full-sequence information was available (see Materials and Methods). The results are shown in figure 4. In agreement with previous analyses (Donnelly et al. 2010; Steinberg et al. 2012), nucleotide diversity estimates were found to be significantly different between the two major haplotypes, with H1 chromosomes showing higher levels of diversity when compared with H2s. Moreover, for both standard and inverted chromosomes, Tajima’s *D* tends to be negative throughout the inversion region.

We simulated neutral inversions in population genetic data to generate theoretical null distributions for both chromosomal types (fig. 4) and found similar significant departures from neutrality for both haplotypes as assessed by Tajima’s *D.*

## Discussion

This study aimed to dissect the complex evolutionary history of the 17q21-*inv* by extending previous efforts to uncharacterized populations, in order to better understand the processes that have shaped the genetic and architectural diversity of this genomic region. In agreement with recent surveys (Stefansson et al. 2005; Zody et al. 2008; Donnelly et al. 2010), we have found that the inversion-associated haplotype family is primarily found in European, Middle Eastern and Southern Asian populations. Furthermore, our analyses revealed that both H2 subtypes are also present in North African populations at appreciable frequencies, with most individuals carrying the duplication-specific version—H2D.

Moreover, by exploring a new set of H2-specific variants, we uncovered considerable genetic diversity levels within the H2 family. This was particularly evident for the H2D subtype, as previous studies had suggested essentially no genetic variation between these chromosomes (Steinberg et al. 2012). Nevertheless, it should be noted that, until now, most estimates were primarily derived from 1) common (nonrandomly selected) polymorphisms, where SNP ascertainment bias could have contributed to the lower overall diversity found within the H2 lineage (Stefansson et al. 2005; Antonacci et al. 2009; Donnelly et al. 2010), and/or 2) resequenced data from a small number of individuals (Steinberg et al. 2012).

In addition, we found that North African H2 subtypes, especially H2′, are genetically closer to other non-African haplotypes than to the ones present in Sub-Saharan Africa. Although extensive gene flow between neighboring populations could have diluted the diversity within the 17q21 region, it has recently been argued (Henn et al. 2012) that North Africans share a common gene pool with populations outside of Africa due to back to Africa movements of populations from the Middle East. As a consequence, North Africans may have diverged more recently from non-African groups than sub-Saharan Africans, thus explaining (at least in part) the observed results. Nevertheless, further work using high throughput sequencing data will be needed to address this question in greater detail.

Also, in agreement with Steinberg et al. (2012), our tree reconstruction indicated the H2′ as the ancestral form from which H2D arose (fig. 3), providing further evidence of an African origin of the H2 haplotype family. In addition, the low levels of differentiation between the remaining H2 chromosomes, as well as the presence of H2′ haplotypes interleaved with H2D branches, suggest that gene flow between the ancestral H2′ subtype and the H2D version may have homogenized the diversity within the inverted segment.

Finally, the nucleotide diversity estimates obtained from resequencing data were substantially different between the two major haplotype families (H1 and H2), with the H1 haplotype showing much higher levels of polymorphism than H2 chromosomes. Moreover, both chromosomal types displayed a negative trend for Tajima’s *D* estimates across the rearrangement, with simulation-based analyses supporting significant departures from neutral expectations. These results, however, should be interpreted with caution as 1) the demographic model simulated is fairly simplistic, and 2) selection cannot be robustly detected using information from a single summary statistic. Also, the fact that the observed data fall tangentially outside the neutral distribution for both orientations suggests that demography may have a stronger contribution than selection to the observed departure. Boettger et al. (2012) have recently argued that the duplicated versions of both major haplotypes (i.e., H1D and H2D) have rapidly increased in frequency in human populations, suggesting that the distinct duplications of *KANSL1* produced a similar phenotype in both backgrounds that could be under selection in modern humans and thus, contrary to previous suggestions (Stefansson et al. 2005; Zody et al. 2008; Donnelly et al. 2010), selection may not be acting on the inversion itself. Unfortunately, given the absence of duplication-tagging markers in the H1 background, and the low number of H2′ individuals available, these suggestions remain merely speculative.

In conclusion, while increasing the known distribution of the inversion associated haplotypes to previously uncharacterized geographical regions, our work showed that the 17q21 H2-family is genetically more diverse and differentiated than initially appreciated (Donnelly et al. 2010; Steinberg et al. 2012). Moreover, our results further highlight the complex structural diversity at 17q21 as sequencing data for both major haplotypes showed evidence of deviation from neutrality. As new and robust methods to analyze complex genome structures emerge, future studies should thus address the possibility of a selective advantage, as well as the phenotypical consequences, of both “derived” haplotypes at the 17q21 locus.

## Supplementary Material

Supplementary material, figures S1–S3, and tables S1–S4 are available at *Genome Biology and Evolution* online (http://www.gbe.oxfordjournals.org/).

Supplementary Data
