# Supplementary material for: Reassessing the Evolutionary History of the 17q21 Inversion Polymorphism
Source: Genome Biol Evol. 2015 Nov 11;7(12):3239–48. doi: 10.1093/gbe/evv214 (PMC4700947; doi:10.1093/gbe/evv214)
Supplement: Supplementary Data [file supp_7_12_3239__index.html]

Reassessing the evolutionary history of the 17q21 inversion polymorphism — Reassessing the Evolutionary History of the 17q21 Inversion Polymorphism — Supplementary Data 

# Reassessing the Evolutionary History of the 17q21 Inversion Polymorphism

## Supplementary Data

files

- Supplementary Data - zip file
