# Supplementary material for: Reassessing the Evolutionary History of the 17q21 Inversion Polymorphism
Source: Genome Biol Evol. 2015 Nov 11;7(12):3239–48. doi: 10.1093/gbe/evv214 (PMC4700947; doi:10.1093/gbe/evv214)

SUPPLEMENTARY INFORMATION

Reassessing the evolutionary history of the 17q21 inversion polymorphism

Joao M Alves*[,1,2,3,4,+], Ana C Lima[1,2,3,5], Isa A Pais[4], Nadir Amir[6], Ricardo Celestino[2,3,7], Giovanna Piras[8], Maria Monne[8], David Comas[9], Peter Heutink[10], Lounès Chikhi[4,11], António Amorim[2,3,12], Alexandra M Lopes[2,3]

1. Doctoral Program in Areas of Basic and Applied Biology (GABBA), University of Porto, Portugal;
2. Instituto de Investigação e Inovação em Saúde, Universidade do Porto, Porto, Portugal – I3S;
3. Instituto de Patologia e Imunologia Molecular da Universidade do Porto, Porto, Portugal – IPATIMUP;
4. Instituto Gulbenkian de Ciência (IGC), Oeiras, Portugal;
5. Department of Genetics, Washington University School of Medicine, St. Louis, MO 63110, USA;
6. Laboratoire de Biochimie Appliquée, Faculté des Sciences de la Nature et de la Vie, Université Abedrrahmane Mira de Bejaia, 06000 Algerie;
7. School of Allied Health Technologies, Polytechnic of Porto, Porto, Portugal;
8. Department of Hematology, Centro di Diagnostica Biomoleculare et Citogenetica Emato-Oncologica, San Francesco Hospital-ASL, Nuoro, Italy;
9. Institut de Biologia Evolutiva (CSIC-UPF), Departament de Ciències Experimentals i de la Salut, Universitat Pompeu Fabra, 08003 Barcelona, Spain;
10. German Center for Neurodegenerative Diseases (DZNE), Tübingen, Germany;
11. CNRS (Centre National de la Recherche Scientifique), Université Paul Sabatier, École Nationale de Formation Agronomique, Unité Mixte de Recherche 5174 EDB (Laboratoire Évolution & Diversit Biologique), F-31062 Toulouse, France;
12. Faculdade de Ciências da Universidade do Porto, Porto, Portugal.

* Corresponding author
Email: jmfernandesalves@gmail.com

Address:
Population Genetics Group, IPATIMUP - Instituto de Patologia e Imunologia Molecular da Universidade do Porto
Rua Dr. Roberto Frias, s/n
4200-465, Porto (PORTUGAL)
Phone: (+351) 22 557 07 00
Fax: (+351) 22 557 07 99

[+] *Current Address:*
Department of Biochemistry, Genetics and Immunology and Institute of Biomedical Research of Vigo (IBIV)
University of Vigo, Vigo
36310, Spain

Cytogenetic validation of 17q21 Inversion by Fluorescent *in situ* Hybridization

Individual lymphoblastoid cell lines were obtained for a total of 5 Hapmap individuals from the Coriell Cell Repository (Camden, NJ USA) (*Supp. Table II*). DUAL-color FISH was performed, using 3 BAC clones (RP11-403G3, RP11-256F16, and RP11-80L9) directly labeled by nick-translation with biotin and digoxigenin, and hybridized to interphase nuclei, as described in (Rao et al. 2010). The cells were then stained with DAPI, and digital images were obtained using a Leica DMRXA2 fluorescence microscope equipped with a CCD camera and appropriate filters. Patterns of fluorescence signals were analyzed for a minimum of 50 interphase nuclei per individual, in order to statistically determine the orientation of the 17q21 segment.

The results are illustrated in *Supp. Figure 1*. A green-red-green (GRG) signal pattern corresponds to the direct orientation, while a green-green-red (GGR) signal pattern corresponds to the inverted orientation. In contrast to (Rao et al. 2010), a perfect correlation between the inversion status and the SNP-defined haplotypes was observed for all individual cell lines. Indeed, the H1 haplotype was found to be associated with the direct orientation while the H2 haplotype was always associated with the inverted configuration.

Consequently, haplotype-informative SNPs (Antonacci et al. 2009, Donnelly et al. 2010) were used to classify the remaining individual samples according to inversion status.

Copy number status validation within the inversion-associated haplotypes

Given that sequence read-depth information may be used to reliably detect genomic regions that differ in copy-number (Sudmant et al. 2010), we assessed the presence/absence of the short *KANSL1* gene duplication (i.e. CNP155) by analyzing the sequence reads from 26 H2 homozygous individuals available from the 1000 Genomes Project (phase III release) (McVean et al. 2012). Copy-number differences were estimated with the HMMCopy software (Ha et al. 2012), following software recommendations. Using one H2 homozygous sample (NA20589), that was previously confirmed by *FISH* to carry the normal copy-number status (*i.e.* H2'/H2') (Steinberg et al. 2012), as control, we individually classified the copy-number status in 18 of the 25 H2D putative homozygous samples. A subset of the results is shown in *Supp. Figure 2*. Interestingly, even with low-coverage re-sequenced data, we were able to classify most individuals according to the copy-

number content of the region. The remaining samples (n=7) had no reads covering the CNP155 region, and were therefore discarded for copy-number assessment. The previously reported duplication-maker SNPs (Steinberg et al. 2012) were thus found to be in perfect correlation with the copy-number content (*Supp. Table II*). These diagnostic SNPs were then used to classify the remaining individuals according to the duplication status.

References

Antonacci F., et al. 2009. Characterization of six human disease-associated inversion polymorphisms. Human Molecular Genetics, 18(14), 2555–66.

Donnelly M.P., et al. 2010. The distribution and most recent common ancestor of the 17q21 inversion in humans. American journal of Human Genetics, 86(2), 161–71.

Ha G, et al. 2012. Integrative analysis of genome-wide loss of heterozygosity and mono-allelic expression at nucleotide resolution reveals disrupted pathways in triple negative breast cancer. Genome Research, 22(10), 1995-2007

McVean G.A., et al. 2012. An integrated map of genetic variation from 1,092 human genomes. Nature, 491, 56-65.

Rao P.N., et al. 2010. Recurrent inversion events at 17q21.31 microdeletion locus are linked to the MAPT H2 haplotype. Cytogenetic and Genome Research, 90095, 275–79.

Steinberg K.M., et al. 2012. Structural diversity and African origin of the 17q21.31 inversion polymorphism. Nature Genetics, 44(8), 872–80.

Sudmant P.H., et al. 2010. Diversity of human copy number variation and multicopy genes. Science, 330, 641–46.

Supplementary Figures & Tables

Supplementary Figure 1. Cytogenetic validation of 17q21-inv on Hapmap cell lines. Fluorescent-signal patterns of the 17q21 segment for 5 Hapmap individuals from distinct continental groups. Green-Red-Green pattern indicates standard orientation; Green-Green-Red pattern indicates inverted orientation. SNP derived haplotypes indicated in each individual digital image. A minimum of 50 interphase nuclei were analyzed for each individual cell line to statistically determine the orientation of the segment.

Supplementary Figure 2. Classification of copy number profiles for the H2-specific CNP155 duplication. Copy-number status derived from read-depth coverage analysis of H2D/H2D (genotype-based) samples a sliding window approach encompassing a region of 400 kb within the 17q21 region. Individual dots represent short non-overlapping genomic segments. Blue dots represent normal copy-number status, while red dots highlight amplification events. Runs of horizontal green lines belong to the same CNV inferred. Dashed delimiters (vertical lines) specify the location of the duplication event at 17q21.

Supplementary Figure 3. Global genetic stratification at the 17q21 region (H2-Specific Panel). Principal Component Analysis (PCA) performed on 852 statistically-derived chromosomes (427 Individual samples carrying at least one copy of the inverted chromosome) from our final data set. A total of 74 SNPs were used. Each dot corresponds to one chromosome, with distinct symbols representing geographical-specific groups. Distinct colors differentiate the two major haplotypes at 17q21 (i.e. H1 and H2). The first principal component (i.e. horizontal axis) illustrates the strong genetic differentiation between the two oppositely oriented haplotypes.

Supplementary Table I. List of Individuals used in the present study.
Supplementary Table II. List of Inversion and Duplication tagging markers.
Supplementary Table III. List of Individuals used in the detection of H2-specific variants.
Supplementary Table IV. List of markers in H2-specific panel.

Supplementary Figure 1.

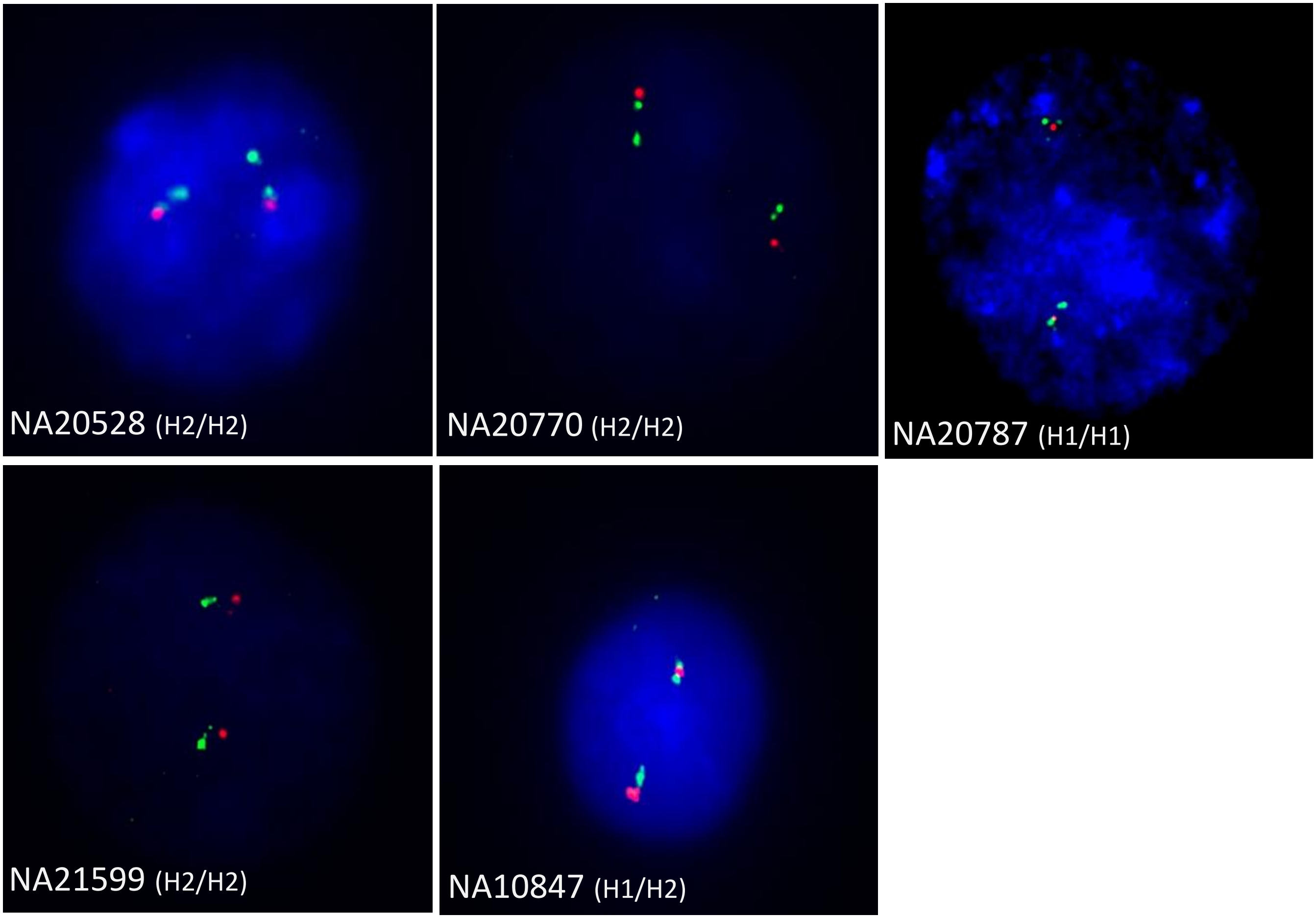

Supplementary Figure 2.

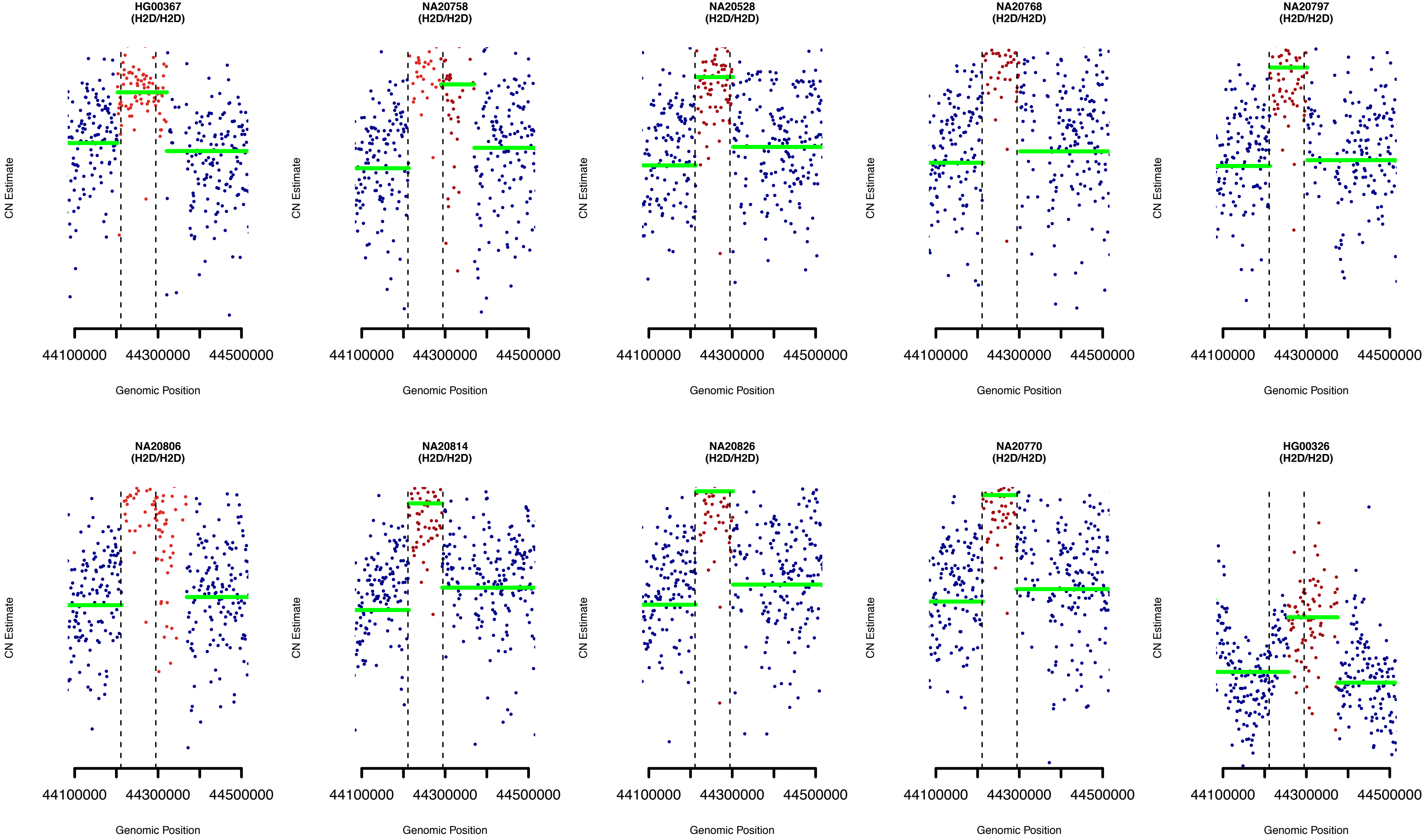

Supplementary Figure 3.

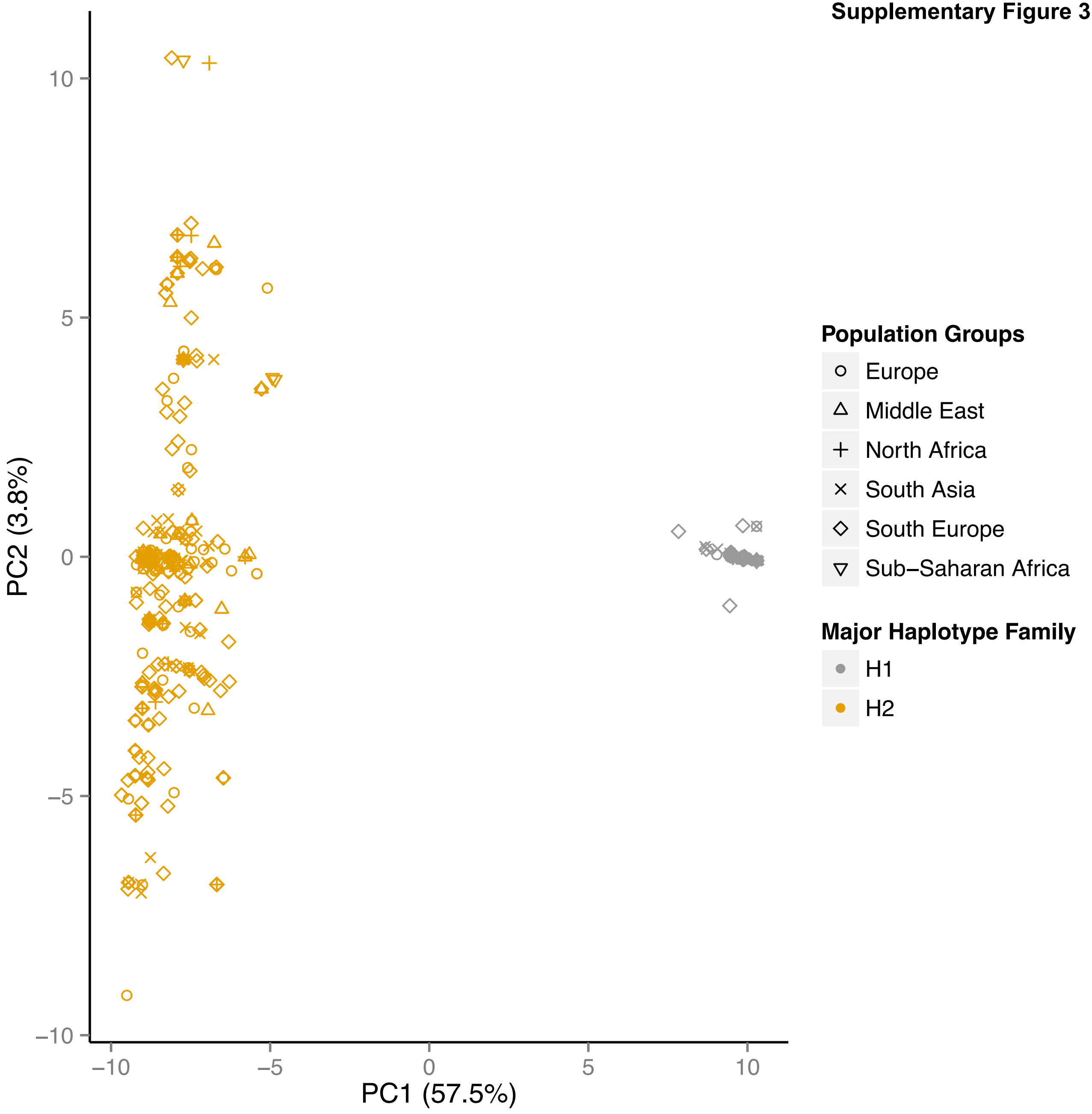

Supplement: Supplementary Data [file supp_evv214_suppl_data.zip › SuppMaterial_Alvesetal_2015_GBE_REVISED.pdf]
