# Supplementary material for: Reassessing the Evolutionary History of the 17q21 Inversion Polymorphism
Source: Genome Biol Evol. 2015 Nov 11;7(12):3239–48. doi: 10.1093/gbe/evv214 (PMC4700947; doi:10.1093/gbe/evv214)
Supplement: Supplementary Data [file supp_evv214_suppl_data.zip › Alvesetal_AdditionalDocument.pdf]

Alves et al. reply to Referee 2:

"… it is still unclear to me the selection of the 26 H2D/H2D individuals that were included in the duplication analysis. According to the methods sentence, "Nevertheless, whenever resequenced data were available (i.e. 1000 Genomes Project data), the presence/absence of the duplication was further confirmed using the HMMCopy software (Ha et al. 2012) (Supp. Info, Supp. Figure 2)", it seems that all available samples were analyzed. However, there are other H2D/H2D homozygotes from 1000GP in Sup. Table 2."

This may be a simple misunderstanding. There are, actually, only 25 H2D/H2D samples as shown in the supplementary table I:

INDIVIDUAL ID	POPULATION GROUP	SOURCE *	FISH-DETERMINED ORIENTATION	SEQUENOM GENOTYPED	READ DEPTH INFORMATION	NEUTRALITY TEST SAMPLES	GENOTYPE
HG00150	EUROPE	1000Genomes			X	X	H2D.H2D
HG00240	EUROPE	1000Genomes			X	X	H2D.H2D
HG00326	EUROPE	1000Genomes			X	X	H2D.H2D
HG00367	EUROPE	1000Genomes			X	X	H2D.H2D
HG01519	SOUTHEUROPE	1000Genomes			X	X	H2D.H2D
HG01531	SOUTHEUROPE	1000Genomes			X	X	H2D.H2D
HG01631	SOUTHEUROPE	1000Genomes				X	H2D.H2D
HG01632	SOUTHEUROPE	1000Genomes			X	X	H2D.H2D
HG01673	SOUTHEUROPE	1000Genomes				X	H2D.H2D
HG01676	SOUTHEUROPE	1000Genomes				X	H2D.H2D
HG01747	SOUTHEUROPE	1000Genomes				X	H2D.H2D
NA12340	EUROPE	1000Genomes			X	X	H2D.H2D
NA20522	SOUTHEUROPE	1000Genomes			X	X	H2D.H2D
NA20528	SOUTHEUROPE	1000Genomes	X	X	X	X	H2D.H2D
NA20587	SOUTHEUROPE	1000Genomes				X	H2D.H2D
NA20758	SOUTHEUROPE	1000Genomes			X	X	H2D.H2D
NA20768	SOUTHEUROPE	1000Genomes			X	X	H2D.H2D
NA20770	SOUTHEUROPE	1000Genomes	X	X	X	X	H2D.H2D
NA20797	SOUTHEUROPE	1000Genomes			X	X	H2D.H2D
NA20806	SOUTHEUROPE	1000Genomes			X	X	H2D.H2D
NA20811	SOUTHEUROPE	1000Genomes			X	X	H2D.H2D
NA20814	SOUTHEUROPE	1000Genomes			X	X	H2D.H2D
NA20826	SOUTHEUROPE	1000Genomes			X	X	H2D.H2D
HG02658	SOUTHASIA	1000Genomes				X	H2D.H2D
NA20890	SOUTHASIA	1000Genomes				X	H2D.H2D

Moreover, only 18 samples (out of the 25) had read-depth information available for copy-

number assessment (but see **Supp. Info** and **Supp. Table I**). We are therefore unsure which H2D/H2D homozygotes the reviewer is referring to.
